# Pancreatic β-cell function is inhibited by miR-3666 in type 2 diabetes mellitus by targeting adiponectin

**DOI:** 10.1590/1414-431X20198344

**Published:** 2019-05-27

**Authors:** J. Tan, A. Tong, Y. Xu

**Affiliations:** 1 Department of Endocrinology, Linyi Central Hospital, Linyi, Shandong, China; 2 Department of Neurology, Linyi Central Hospital, Linyi, Shandong, China

**Keywords:** Type 2 diabetes mellitus, miR-3666, Insulin secretion, Cell proliferation, ADIPOQ

## Abstract

Type 2 diabetes mellitus (T2D) is a common endocrine and metabolic disorder, and poses threats to human health worldwide. Recently, microRNAs (miRNAs) have been suggested to play important roles in the pathophysiology of T2D. In this study, we explored the role of miR-3666 in T2D. miR-3666 was significantly down-regulated in the serum of T2D patients when compared to that of healthy volunteers, and miR-3666 expression level was negatively correlated with blood glucose levels of T2D patients. Overexpression of miR-3666 inhibited cell proliferation, reduced insulin secretion, and promoted cell apoptosis of pancreatic β-cell line (INS-1 cells). On the other hand, knockdown of miR-3666 had the opposite effects in INS-1 cells. The bio-informatics analysis using TargetScan revealed that adiponectin (ADIPOQ) was a downstream target of miR-3666, and the interaction between miR-3666 and ADIPOQ was validated by luciferase reporter assay. In addition, miR-3666 negatively regulated the mRNA and protein expression of ADIPOQ. Overexpression of ADIPOQ promoted insulin secretion after glucose stimulation, promoted cell proliferation, inhibited cell apoptosis, and partially abolished the effects of miR-3666 overexpression on insulin secretion, cell proliferation, and cell apoptosis of INS-1 cells. In conclusion, our results revealed that miR-3666 inhibited pancreatic cell proliferation, reduced insulin sensitivity, and promoted apoptosis by targeting ADIPOQ.

## Introduction

Type 2 diabetes mellitus (T2D) is a disease affecting about 500 million people worldwide, and incidence of T2D is expected to increase (1,2). T2D is a common endocrine and metabolic disorder characterized by pancreatic β-cell dysfunction in response to elevated blood glucose levels, insulin resistance, and the failure of peripheral tissues in response to physiological levels of insulin (3,4). The yearly costs of preventing and managing diabetes are extremely high. However, the molecular mechanisms underlying the pathogenesis of T2D are not fully elucidated. Thus, the understanding of the exact mechanisms of T2D pathogenesis and the development of effective therapy are of great scientific interest.

MicroRNAs (miRNAs) are a class of small, non-coding RNAs with ∼22 nucleotides in length that act to inhibit translation or directly induce degradation by binding to specific complementary sites within 3′ untranslated region (3′UTR) of targeted genes (5). A large number of studies have shown the functional role of miRNAs in the development of various diseases, including cancer, neurodegenerative diseases, cardiovascular diseases, and diabetes (6–8). Recently, many studies have revealed the critical roles of miRNAs in pancreatic development and function related to the pathogenesis of T2D. For example, miR-144 impairs insulin signaling by inhibiting the expression of insulin receptor substrate 1 in T2D (9). Frost et al. (10) showed that miRNA Let-7 regulated multiple aspects of glucose metabolism and insulin sensitivity and suggested that knockdown of Let-7 might represent a potential treatment for T2D. miR-200 family is strongly induced in islets of diabetic mice and miR-200 overexpression is sufficient to induce β-cell apoptosis and lethal T2D, suggesting the crucial role for the miR-200 family in β-cell survival and the pathophysiology of diabetes (11). miR-19a-3p enhances the proliferation of pancreatic β-cells and insulin secretion, and inhibits apoptosis of pancreatic β-cells via targeting SOCS3 (12). Recently, studies found that miR-3666 was up-regulated in many cancers, its overexpression induced cancer cell proliferation and migration, and inhibited cancer cell apoptosis (13–16). The role of miR-3666 has been extensively examined regarding the pathophysiology of different types of cancers (17). However, the role of miR-3666 in the pathogenesis of T2D has not been examined.

In the present study, we identified the expression of miR-3666 in the peripheral blood of T2D patients and healthy volunteers. *In vitro* studies were further performed to elucidate the functional role of miR-3666 in cell proliferation, insulin sensitivity, and cell apoptosis of pancreatic β-cells. Our findings may reveal a novel role of miR-3666 in the pathophysiology of T2D.

## Material and Methods

### Blood sample collection

Blood samples were collected from 60 patients with T2D and 30 healthy volunteers in the Linyi Central Hospital from July 2017 to July 2018. The World Health Organization criteria for T2D diagnosis (18) are: a) random blood glucose ≥200 mg/dL, b) glycosylated hemoglobin ≥6.5%, c) blood glucose after oral 75 g glucose overload ≥200 mg/dL, and d) 8-h fasting glucose ≥110 mg/dL. Patients with malignancy, acute heart failure, or severe kidney or liver diseases were excluded from this study. This study was approved by the Ethics Committee of Linyi Central Hospital, and was carried out in accordance with the Declaration of Helsinki. Written informed consent was obtained from all subjects prior to blood collection.

### Cell culture

The INS-1 cell line (ATCC, USA) was cultured in Dulbecco's modified Eagle's medium containing high glucose (Thermo Fisher Scientific, USA), supplemented with 10% fetal bovine serum (FBS, Thermo Fisher Scientific) and 70 μM β-mercaptoethanol (Sigma, USA) at 37°C with 5% CO_2_ in a humidified incubator.

### Oligonucleotides and cell transfection

The miRNAs including miR-3666 mimic (miR-mimic), miR-3666 inhibitor (miR-inhibitor), and their respective negative controls (mimic-NC and inhibitor-NC) were purchased from RiboBio (China). The control vector (pcDNA3.1) and the adiponectin (ADIPOQ)-overexpressing vector (pcDNA3.1-ADIPOQ) were obtained from GenePharma (China). Cell transfection or co-transfection was performed by using Lipofectamine 2000 reagent (Invitrogen, USA) according to the manufacturer's protocol. At 48 h after transfection, INS-1 cells were collected for further analysis.

### RNA extraction and quantitative real-time PCR (qRT-PCR)

Total RNA from peripheral blood (serum) or cells were extracted using TRIzol reagent (Invitrogen) according to the manufacturer's protocol. The relative expression was determined using the mirVana qRT-PCR detection kit (Thermo Fisher Scientific) according to the manufacturer's instructions. The relative mRNA expression of ADIPOQ was detected using the standard SYBR-Green real-time PCR kit (Takara, China) according to the manufacturer's instructions. U6 was used as an internal reference for miR-3666, and GAPDH was used as an internal reference for ADIPOQ mRNA expression. The relative expression levels were quantified using the comparative Ct method.

### Measurement of glucose-stimulated insulin secretion

INS-1 cells were seeded in a 96-well plate with further culturing for 24 h, and the cells were treated with 3.3 mM glucose (basal glucose) or 16.7 mM glucose (stimulatory glucose) for 1 h. After that, the insulin level was measured by Insulin ELISA kit (Abcam, UK) according to the manufacturer's instructions.

### Cell viability assay

Transfected INS-1 cells were seeded in a 96-well plate with further culturing for 24 h. After that, cells were incubated with 20 μl of MTT solution (5 mg/mL) for 4 h at room temperature, and then 200 μL of DMSO was added to each well to dissolve the formazan for 10 min at room temperature. The cell viability was detected by measuring the absorbance at 490 nm using a microplate reader (BioTek, USA) according to the manufacturer's instructions.

### Flow cytometry to detect cell apoptosis

The cell apoptotic rate was measured using the annexin V-FITC apoptosis detection kit (Thermo Fisher Scientific) according to the manufacturer's instructions. Transfected cells were collected, washed in PBS, resuspended in binding buffer containing annexin-FITC and propidium iodide, and incubated for 15 min at room temperature in the dark. Cell apoptosis of INS-1 cells was analyzed using a FACScalibur flow cytometer (BD Biosciences, USA).

### Dual-luciferase reporter assay

The target relationship between miR-3666 and ADIPOQ was predicted by TargetScan database (<http://www.targetscan.org>, Whitehead Institute, USA). The pmiRGLO vector was used to construct the wild type and mutated type 3′UTR of ADIPOQ. miR-3666 mimic or mimic-NC and wild type or mutated type of ADIPOQ 3′UTR were co-transfected into INS-1 cells using Lipofectamine 2000 reagent (Invitrogen) according to the manufacturer's instructions. At 48 h after transfection, luciferase activities were quantified by Dual-Luciferase reporter assay (Promega, USA).

### Western blot assay

Cells were lysed with RIPA buffer containing protease inhibitors (Sigma), and the extracted protein levels were determined by BCA method (Bio-Rad, USA). The proteins were separated on a 10% SDS-polyacrylamide gel and transferred onto polyvinylidene difluoride membranes (PVDF; Millipore, USA). The PVDF membrane was blocked with 5% skim milk for 1 h at room temperature. After that, the PVDF membrane was further incubated with primary antibodies against ADIPOQ and β-actin (Cell Signaling Technology, USA) at 4°C overnight. After incubation with horseradish peroxidase-conjugated secondary antibodies for 1 h at room temperature, the blots were visualized by ECL reagent (Thermo Fisher Scientific).

### Statistical analysis

All data are reported as means±SE, and GraphPad Prism 6 software (USA) was used to plot graphs and perform statistical analysis. The association between miR-3666 expression level and blood glucose level was analyzed using Spearman correlation analysis. Differences were analyzed using Student's *t*-test or one-way ANOVA. P<0.05 was considered statistically significant.

## Results

### miR-3666 was down-regulated in the peripheral blood of T2D patients

**Figure 1:**
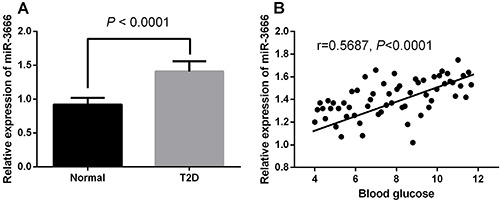
**A**, miR-3666 was up-regulated in the peripheral blood of type 2 diabetes mellitus (T2D) patients (measured by qRT-PCR in peripheral blood from 30 healthy volunteers and 60 patients with T2D). Data are reported as means±SE, and were compared with the *t*-test. **B**, Correlation between miR-3666 level and blood glucose determined by Spearman correlation analysis (r=0.5687, P<0.0001).

The anthropometric and clinical variables of subjects were as follow: 45.81±5.92 years of age, 36 men and 24 women, BMI=25.12±0.31 kg/m^2^, glucose=175.34±19.91 mg/dL, cholesterol=228.65±31.15 mg/dL, triglycerides=163.63±21.82 mg/dL, HDL-c=51.02±3.43 mg/dL, and LDL-c=121.12±21.02 mg/dL. The qRT-PCR assay showed that miR-3666 was significantly up-regulated in the serum of T2D patients compared with that of healthy volunteers (Figure 1A). Furthermore, the expression level of miR-3666 was negatively correlated with blood glucose level (Figure 1B).

### Effects of miR-3666 on the insulin secretion, cell proliferation, and apoptosis of pancreatic β-cells

**Figure 2:**
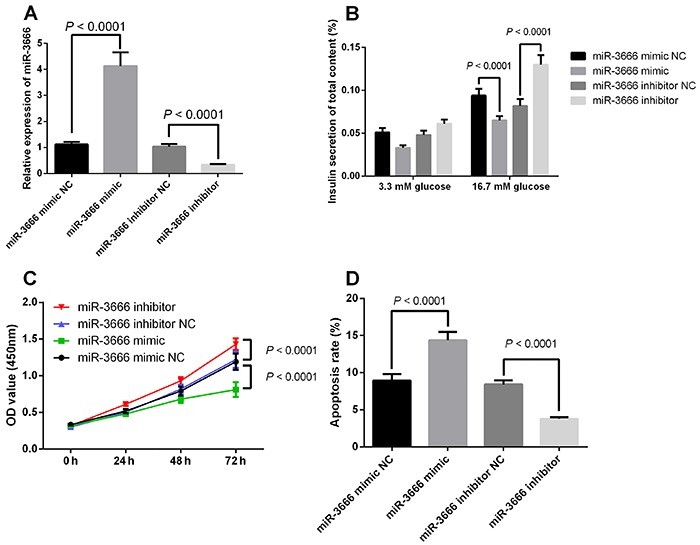
Effects of miR-3666 on the insulin secretion, cell proliferation and apoptosis of pancreatic β-cells. INS-1 cells were transfected with mimic-normal control (NC), miR-3666 mimic inhibitor-NC, or miR-3666 inhibitor. **A**, Relative expression of miR-3666. **B**, Glucose-stimulated insulin secretion was determined by ELISA assays. **C**, Cell proliferation was determined by CCK-8 assay. **D**, Cell apoptosis was determined by flow cytometry. Data are reported as means±SE (n=3) and were compared with the *t*-test or ANOVA.

As shown in Figure 2A, INS-1 cells transfected with miR-3666 mimic had significantly higher expression levels of miR-3666 than those transfected with mimic-NC, and transfection with miR-3666 inhibitor suppressed the expression of miR-3666 in the INS-1 cells. The ELISA assay showed that insulin secretion in response to glucose stimulus was decreased in cells transfected with miR-3666 mimic (Figure 2B) and increased in cells transfected with miR-3666 inhibitor (Figure 2B). The MTT assay showed that overexpression of miR-3666 decreased cell proliferative ability of INS-1 cells (Figure 2C), and knockdown of miR-3666 promoted cell proliferation (Figure 2C). In addition, flow cytometry results showed that overexpression of miR-3666 promoted cell apoptosis of INS-1 cells (Figure 2D), and knockdown of miR-3666 inhibited cell apoptosis of INS-1 cells (Figure 2D).

### ADIPOQ was a direct target of miR-3666 in pancreatic β-cells

**Figure 3:**
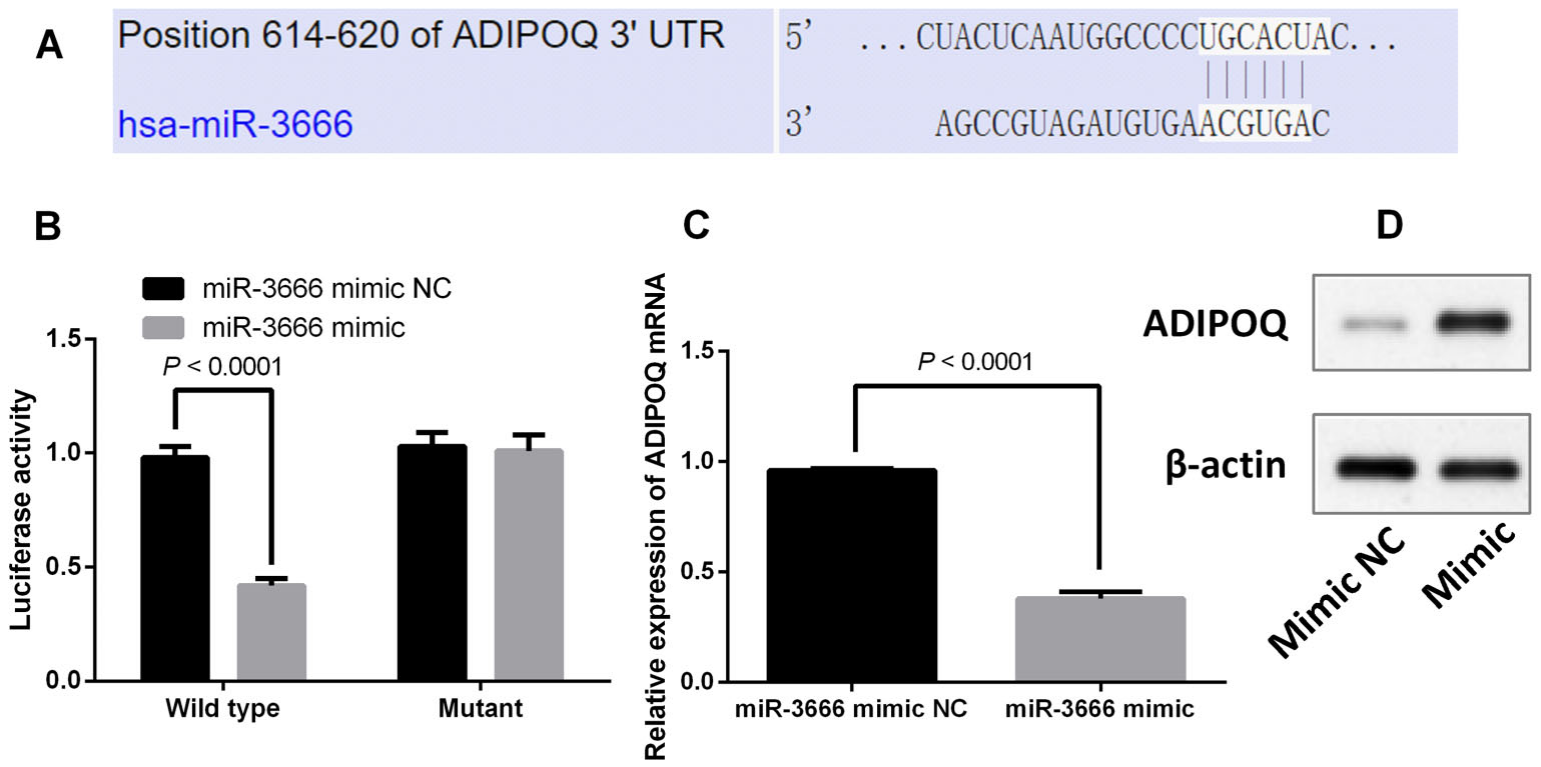
Adiponectin (ADIPOQ) is a direct target of miR-3666 in pancreatic β-cells. **A**, Interaction sites between miR-3666 and 3′UTR of ADIPOQ. **B**, INS-1 cells were co-transfected with firefly luciferase constructs containing the ADIPOQ wild type or mutant 3′UTR and miR-3666 mimic (miR-mimic) or mimic-normal control (NC). Luciferase activity was measured 48 h after transfection. **C** and **D**, mRNA levels and protein expression of ADIPOQ after transfection with miR-mimic or mimic-NC. Data are reported as means±SE (n=3) and were compared with the *t*-test.

miRNA regulated gene expression via targeting the 3′UTR, and the predicted targets of miR-3666 were identified using TargetScan software. ADIPOQ was one of the potential targets of miR-3666. The interaction sites between miR-3666 and 3′UTR ADIPOQ are shown in Figure 3A. To further validate the interaction between miR-3666 and ADIPOQ, we cloned the 3′UTR of ADIPOQ segment (wild type or mutant) into the luciferase reporter vector. Transfection with miR-3666 mimic suppressed the luciferase activities of the wild type 3′UTR of ADIPOQ, but the construct containing a mutant binding site abolished the inhibitor effect of miR-3666 overexpression (Figure 3B). Furthermore, qRT-PCR and western blot results showed that miR-3666 overexpression suppressed the mRNA and protein expression of ADIPOQ (Figure 3C and D).

### ADIPOQ was involved in the miR-3666-mediated effects on pancreatic β-cells functions

**Figure 4:**
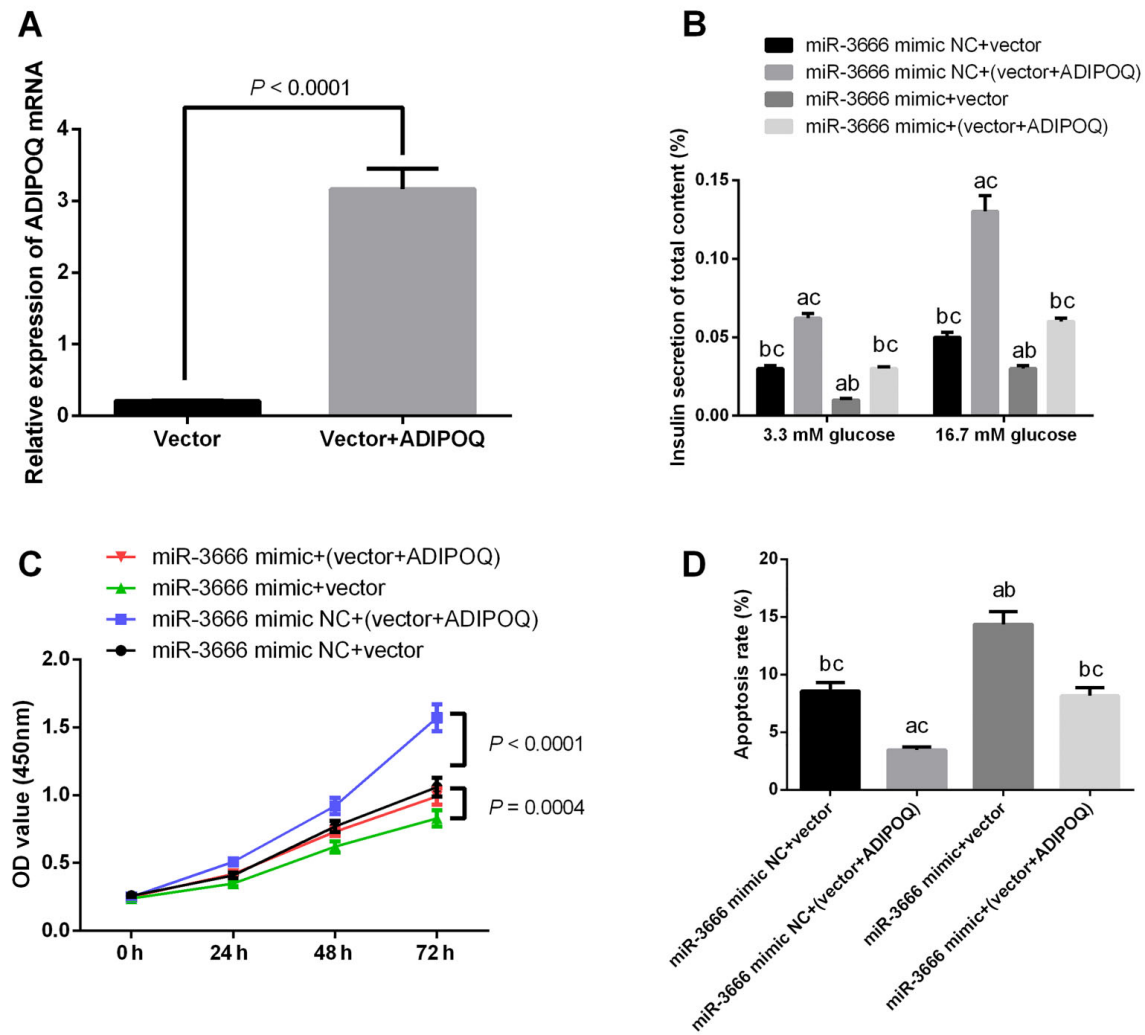
ADIPOQ was involved in the miR-3666-mediated effects on the functions of pancreatic β-cells. **A**, INS-1 cells were transfected with pcDNA3.1 (vector) or pcDNA3.1-ADIPOQ (ADIPOQ), and the mRNA expression of ADIPOQ was determined by qRT-PCR. INS-1 cells were co-transfected with pcDNA3.1-ADIPOQ (or pcDNA3.1 vector) and miR-3666 mimic (or mimic-normal control (NC)). **B**, Glucose-stimulated insulin secretion was determined by ELISA assay. **C**, Cell proliferation was determined by MTT assay. **D**, Cell apoptosis was determined by flow cytometry. ^a^P<0.05 compared to miR-3666 mimic NC+vector; ^b^P<0.05 compared to miR-3666 mimic NC+(vector+ADIPOQ); ^c^P<0.05 compared to miR-3666 mimic+vector. Data are reported as means±SE (n=3) and were compared with the *t*-test or ANOVA.

To further examine the relevance of miR-3666/ADIPOQ axis in the regulation of pancreatic β-cells, INS-1 cells were transfected with ADIPOQ overexpression vector (pcDNA3.1- ADIPOQ; Figure 4A). As shown in Figure 4B–D, overexpression of ADIPOQ promoted insulin secretion in glucose stimulation, promoted cell proliferation, inhibited cell apoptosis, and enforced expression of ADIPOQ. In addition, the transfection partially abolished the effects of miR-3666 overexpression on insulin secretion, cell proliferation, and cell apoptosis of INS-1 cells.

## Discussion

T2D is one of the most severe threats to human health worldwide. Recently, various miRNAs such as let-7, miR-7, miR-9, and miR-21 have been linked to the pathogenesis of T2D (19). In our current study, we showed the up-regulation of miR-3666 in the peripheral blood of T2D patients, and the miR-3666 expression level was negatively correlated with blood glucose levels of T2D patients. The *in vitro* functional studies revealed that miR-3666 decreased insulin sensitivity, inhibited cell proliferation, and induced cell apoptosis of pancreatic β-cells. The bioinformatics analysis and luciferase reporter assay further identified ADIPOQ as a downstream target of miR-3666, and ADIPOQ was involved in the effects of miR-3666 overexpression on insulin sensitivity, cell proliferation, and cell apoptosis of pancreatic β-cells.

miRNAs play a critical role in carcinogenesis of various tumors as either tumor suppressor or enhancer. Among all miRNAs, miR-3666 has been rarely studied and reports on its involvement in cancer are lacking. Previous studies reported that miR-3666 was up-regulated in colorectal cancer, lung cancer, thyroid cancer, and cervical cancer tissues, and the down-regulation of miR-3666 could significantly inhibit the development of tumors (13–16). Consistently, our results revealed that miR-3666 was down-regulated in the serum of T2D patients and was negatively correlated with blood glucose levels, suggesting that down-regulation of miR-3666 played a role in the pathophysiology of T2D. Our functional studies further revealed that overexpression of miR-3666 increased insulin sensitivity, promoted cell proliferation, and inhibited cell apoptosis of pancreatic β-cells, suggesting that miR-3666 was important in attenuating pancreatic β-cell dysfunction in T2D.

The bioinformatics analysis showed that ADIPOQ was a potential target of miR-3666. Adiponectin (also known as Acrp30 (20), AdipoQ (21), GBP-28 (22), and apM1 (23)) is a 244-amino acid protein secreted mainly by adipose tissue. It was identified almost simultaneously by four different groups using different approaches (20). Initially, it was thought that adiponectin was exclusively produced by adipose tissue. Later, different research groups reported that adiponectin is expressed in other tissues including human and murine osteoblasts (24), liver parenchyma cells (25), myocytes (26), epithelial cells (27), and placental tissue (28). Human adiponectin is encoded by the AdipoQ gene, which spans 17 kb on chromosome locus 3q27. The gene for human adiponectin contains three exons, with the start codon in exon 2 and stop codon in exon 3 (29). This human chromosome 3q27 has been identified as a region carrying a susceptibility gene for T2D and metabolic syndrome (30). Serum levels of adiponectin decrease with obesity and are positively associated with insulin sensitivity (31). Adiponectin is a 30 kDa multimeric protein and is secreted mainly by white adipose tissue, although other tissues also express low levels of adiponectin. Full-length human adiponectin comprises 244 amino acid residues, including a NH2-terminal hyper-variable region (amino acids from 1–18), followed by a collagenous domain consisting of 22 Gly-XY repeats, and a COOH-terminal C1q-like globular domain (amino acids from 108–244) (32). Collectively, the results of our study suggest that miR-3666-mediated effects on pancreatic β-cell function were partially via modulating ADIPOQ.

In conclusion, our results showed that patients with T2D presented higher expression level of miR-3666 in serum, and miR-3666 expression level was negatively associated with blood glucose levels. *In vitro* studies revealed that miR-3666 inhibited pancreatic cell proliferation, reduced insulin sensitivity, and promoted apoptosis by targeting ADIPOQ. Our results suggested the potential therapeutic role of miR-3666 in T2D. However, our study focused on a single target gene of ADIPOQ, the signaling pathways that regulate T2D are still unclear, and the effect of the pharmacological treatment based on miR-3666 expression remains to be explored.
